# Biological Control of Plant Diseases: An Evolutionary and Eco-Economic Consideration

**DOI:** 10.3390/pathogens10101311

**Published:** 2021-10-12

**Authors:** Dun-Chun He, Meng-Han He, Divina M. Amalin, Wei Liu, Dionisio G. Alvindia, Jiasui Zhan

**Affiliations:** 1 Institute of Eco-technological Economics, School of Economics and Trade, Fujian Jiangxia University, Fuzhou 350108, China; hedc@fjjxu.edu.cn; 2 College of Plant Protection, Henan Agricultural University, Zhengzhou 450002, China; hemenghan@henau.edu.cn; 3 Department of Biology, De La Salle University, Manila 1004, Philippines; divina.amalin@dlsu.edu.ph; 4 Center for Natural Science and Environmental Research, De La Salle University, Manila 1004, Philippines; boyet.alvindia@da.gov.ph; 5 School of Life Science, Ningde Normal University, Ningde 352100, China; t9703@ndnu.edu.cn; 6 Philippine Center for Postharvest Development and Mechanization, Nueva Ecija 3120, Philippines; 7 Department of Forest Mycology and Plant Pathology, Swedish University of Agricultural Sciences, 75007 Uppsala, Sweden

**Keywords:** pathogen evolution, sustainable plant disease management, ecological resilience, economic benefit, functional tradeoffs, social involvement, microbiome, plant immunity

## Abstract

Biological control is considered as a promising alternative to pesticide and plant resistance to manage plant diseases, but a better understanding of the interaction of its natural and societal functions is necessary for its endorsement. The introduction of biological control agents (BCAs) alters the interaction among plants, pathogens, and environments, leading to biological and physical cascades that influence pathogen fitness, plant health, and ecological function. These interrelationships generate a landscape of tradeoffs among natural and social functions of biological control, and a comprehensive evaluation of its benefits and costs across social and farmer perspectives is required to ensure the sustainable development and deployment of the approach. Consequently, there should be a shift of disease control philosophy from a single concept that only concerns crop productivity to a multifaceted concept concerning crop productivity, ecological function, social acceptability, and economical accessibility. To achieve these goals, attempts should make to develop “green” BCAs used dynamically and synthetically with other disease control approaches in an integrated disease management scheme, and evolutionary biologists should play an increasing role in formulating the strategies. Governments and the public should also play a role in the development and implementation of biological control strategies supporting positive externality.

## 1. Introduction

Plant diseases caused by infectious pathogens have seriously affected human society and nature through their damages to food production, economic development, ecological resilience, and natural landscapes over human history. Hunger and malnutrition of the Irish famine [1] caused by the potato late blight pathogen *Phytophthora infestans* (Mont.) de Bary, and the Bengali famine [2], caused by the rice brown spot pathogen *Bipolaris oryzae* (Breda de Haan) Shoemaker, led to millions of deaths and uprooted families and social structures. The pandemics of chestnut blight caused by *Cryphonectria parasitica* (Murrill) Barr [3] and Dutch elm disease caused by *Ophiostoma novo-ulmi* (Buism.) Nann. [4] destroyed a large proportion of primary and secondary forestry in North America and Europe, leading to ecological catastrophe in the regions. In addition, many plant pathogens produce mycotoxins that directly or indirectly threaten the health of humans and animals [5].

Plant diseases can occur in the entire crop production chain and remain as one of the greatest threats to the sustainable development of society, resulting in a 13%–22% annual yield loss, or billions of US dollar economic costs in staples of rice, wheat, maize, and potato along with additional costs spent on education and the development of management strategies [6,7]. These biological and economic losses at least partially account for the recent estimates of ~800 million people in the world experiencing starvation or undernourishment [7,8].

## 2. Approaches of Plant Disease Control

Plant diseases result from complex interactions among plants, pathogens, and the environment. In the long history of agriculture, humans have developed a variety of approaches to manipulate the interaction to create a system in favor of the growth and development of host plants but suboptimum to the establishment, reproduction, and transmission of pathogens [9]. Depending on circumstances of crop, pathogen, geographic location, technology availability, regulation policy, and other factors, these control approaches can be agronomic (e.g., crop diversification and field hygiene), regulative (e.g., quarantine and eradication), genetic (e.g., disease resistance and tolerance), physical (e.g., soil solarization and flooding), and chemical (e.g., pesticides and host-immunity inducer) and can be used individually or in combination (integrated disease management, IDM) to suppress causal pathogen, promote host immunity, or change the biotic and abiotic environment where host–pathogen interaction occurs.

Among these control strategies, host resistance is one of the most economic and eco-friendly methods to control plant diseases. In the vertical resistance mediated by gene-for-gene interaction such as in wheat (*Puccinia triticina* Eriks) and potato (*P. infestans*) systems [10,11], resistant responses in plants are triggered by a small group of proteins (effectors) secreted from pathogens which were recognized by the corresponding receptor proteins produced by host resistance genes [12,13]. This resistance is complete but can be easily evaded due to the continuous evolution of pathogens, leading to rapid breakdown of resistant varieties after they are released for commercial utilization [14]. In some cases, resistance can be broken down even when it was deployed in a limited acreage, such as grapevine resistance to downy mildew [15]. This is particularly a problem in modern agriculture, which is associated with intensification and monoculture that create the most conducive conditions for pathogen evolution [16,17]. Horizontal or partial resistance is governed by many genes which distribute over plant genomes and regulate many biological, biochemical, and ecological processes of cells [18]. The resistance is incomplete and often requires additional disease control approaches to ensure better harvest but is more durable compared to vertical resistance due to the minor and accumulative contribution of each gene to the resistant phenotypes [19].

When host resistance is unavailable or insufficient to suppress disease epidemics, fungicide application becomes inevitable. In the philosophy of free-disease agriculture currently adopted worldwide, fungicides are often overused to guarantee crop yield and quality, particularly for vegetable and ornamental productions in developed regions. For example, as many as 20 fungicide applications are executed in European potato production [20], even though some of these applications do not necessarily generate a further biological or economic return. In addition to financial cost and the risk of reducing efficacy, widescale and inappropriate applications of fungicides can cause environmental problems due to their negative effects on soil and water quality, biodiversity, and animal and human health [21]. It is documented that >70% of apple orchards in Shanxi and Shandong provinces, China, are experienced with the excessive use of pesticides [22]. 

Biological control is a method of plant disease management by inhibiting plant pathogens, improving plant immunity, and/or modifying the environment through the effects of beneficial microorganisms, compounds, or healthy cropping systems [23,24,25,26,27,28,29,30]. Biological control offers several advantages over other approaches of plant disease management by taking into consideration the following: (1) biological control agents (BCAs) usually target a specific group of pathogens and therefore have fewer negative impacts on the ecosystem as opposed to fungicides, even though some risks of ecological issues (as described in Section 4.3) should be considered, particularly with the introduction of non-native species [31,32]; (2) many BCAs can sustain themselves and keep in place for a longer time without additional efforts to keep the system running [33,34]. For example, long-term effects of BCAs *Trichoderma harzianum* Rifai, *Pochonia chlamydosporia* (Goddard) Zare and Gams, and *Paecilomyces lilacinus* (Thom) Samson to suppress soybean root diseases have been reported in Northeast China [34]; and (3) a documented tradeoff exists between host resistance and agronomic traits [35]. BCAs prevent the consistent expression of the host immune system, allowing plants to allocate more energy and resource for agronomic traits important to farmers [36]. To date, despite the well-known documentation of biological control as an important component of IDM, its commercial value is less than 5% of the total crop protection market [37,38]. The low commercial contribution is highly associated with low technology transfer such that its economic value is not yet realized by the agricultural community, particularly in developing countries. The efficacy of many BCAs is usually strongly affected by biotic and abiotic factors, and its durability under continuous pathogen evolution is concerned, further constraining the application of the approach. Addressing this dilemma requires a better understanding of the interaction of BCAs with plants, pathogens, and the environment in the context of economics, ecology, and evolution.

## 3. Types and Mechanisms of Biological Control

As a promising approach to plant disease management, the concept of biological control is dated to 4000 years ago in Egypt [39]. However, the advanced study of biological control did not start until the nineteenth century [40]. The discovery that the severity of some soil-borne diseases was mitigated by *Bacillus subtilis* (Ehrenberg) Cohn, *Ampelomyces quisqualis* Ces, and other antagonistic microorganisms stimulated the exploration of using BCAs to manage plant diseases [41,42]. Since then, research in biological control has been revolutionized. A great number of BCAs have been developed, including the utilization of beneficial microorganisms [43], plant inducers, microbial metabolites, and plant extracts in-crop diversification [44,45,46,47,48]. According to their modes of action, these BCAs can be divided into three categories, as discussed below.

### 3.1. Suppressing Pathogens

Some microbes are hyperparasites that produce antibiosis to directly kill pathogens or rely on pathogens for energy supply or living environments, while others may serve as competitors for niche and nutrients by releasing compounds or antimicrobials [49,50,51]. Some fungi, mycoviruses, and bacteriophages have these properties. They can potentially be BCAs augmented against plant pathogens and applied in fields once or several times depending on their biological features and environments [52,53]. The secondary metabolites and compounds released by microbial or non-microbial species can also be used as pathogen inhibitors to control plant diseases. Plants can defend themselves by producing compounds to kill pathogens or promote the growth of beneficial microbes [54]. These compounds can be extracted from plants and used in combination with antimicrobials or metabolism produced by beneficial microbes such as BCAs [55,56]. For example, many bacterial and fungal endophytes produce myriad secondary metabolites that have antagonistic, inhibitory, and deterrent properties that defer plant pathogens [57,58]. The antibiosis of endophytic BCAs is triggered by different types of secondary metabolites they produce [59]. *Pseudozyma flocculosa* produces a compound that induces a rapid formation of cell collapse in the pathogen and is an effective BCA to control powdery mildew [60]. *Pseudomonas chlororaphis* (Guignard and Sauvageau) Bergey produces phenazines, pyrrolnitrine, 2-hexyl, 5-propyl resorcinol and hydrogen cyanide, siderophores, and a complex blend of volatile organic compounds that effectively contribute to the control of several plant pathogens and nematodes [61]. *Fluorescent pseudomonads* has been used to compete with several pathogenic fungi and bacteria [62]. Some strains of *Bacillus* sp. deliver antagonizing metabolites into the root system, where they directly suppress the growth of pathogenic bacteria [63]. *Orthotydeus lambi* Baker and epiphytic yeasts have been successfully used to control grape [64] and cucurbit powdery mildew [65]. *B. subtilis* GLB191 is also used against the biotrophic oomycete *Plasmopara viticola* (Berk. and Curt.) Berl and de Toni, the causal agent of grapevine downy mildew [66]. Serenade produced from a specific strain of *B. substilis* QRD137 suppresses floral infection and subsequent growth of a pathogen in flowers of blueberries [67]. Some other antagonist microorganisms were also found to inhibit the growth of pathogens, such as *Erwinia chrysanthemi* Burkholder, causing tomato bacterial stem rot [68]. In addition to antagonism, biological control can also be achieved by using avirulent strains of pathogen species. A classical example of within-pathogen-species competition is from the release of avirulent *Aspergillus flavus* Link genotypes, which reduces the contamination of cotton and other crops by aflatoxin [69].

### 3.2. Compounds Priming, Inducing, or Strengthening Plant Defense Responses

Some beneficial microbes interact with plants to induce host resistance or prime host immunity responses without direct contact with pathogens [70,71]. These agents include the natural products and chemical compounds produced by different sources, such as plant extracts, microbial metabolites, synthetic chemicals, and gene products [72]. Many secondary metabolites involved in signal transduction, catalytic activities, and compounds such as salicylic acid, acetylsalicylic acid, and nitric oxide have properties that induce host plant immunity and enhance host resistance [73]. These compounds are responsible for the observed systemic acquired resistance after host plants are infected by pathogens [74] and can be produced by many other non-pathogenic microbes, such as rhizobacteria [75]. They are also commonly found in plant tissues but vary widely in extent among species, even genotypes within the same species [73]. It is evident that some of these inducer compounds not only suppress plant diseases but also improve plant vigor, possibly due to the enhanced production of hormones [76]. For example, *T. harzianum* produces a butenolide metabolite called harzianolide that stimulates growth and defense mechanisms of tomato plants, resulting in a 16–30% reduction of disease caused by *S. sclerotiorum* [77]. Attempts to induce an immune response against *P. infestans* by treating potatoes with various fatty acids have achieved 39–82% protection [78]. Similarly, root rot diseases of green beans caused by *Fusarium solani* Marti and *Rhizoctonia solani* Kühn were substantially suppressed (60–80%) after field treatments of chitosan salicylic acid and humic acid [45]. However, the exploitation and utilization of the active substances for BCAs for commercial use is usually costly and low efficient partially due to the time lag of inducing plant resistance [79].

### 3.3. Regulating the Ecosystem to Protect and Promote Natural Enemies or Competitors of Pathogens

Plant disease often results from a disordered ecosystem [80]. The success of biological control relies on a healthy ecosystem provided by predators, competitors, promoters, and other species. These beneficial organisms have spatiotemporal dynamics in crop fields as the function of genetics, composition, and structure of local plant and microbial communities [81]. The beneficial interplay of the microbiome with other organisms in soil communities is particularly important in maintaining a functional ecosystem for the growth and immunity development of plants. Methanol can suppress the growth of methanotrophs that can survive by coexisting with *Hyphomicrobium* spp. to build a rhizospheric microbial association, in which *H.* spp. is capable of improving effective nutrient utilization and removing harmful methanol in the rhizosphere [82]. One attempt in biological control is to improve environmental quality by increasing the amount and diversity of beneficial microorganisms in farmlands to suppress the occurrence and development of pathogens, which can be achieved through crop diversification such as crop rotation, intercropping, and cultivar mixture. There is increasing evidence showing that crop diversification can suppress plant diseases [83,84]. Disease suppression by crop diversification involves multiple mechanisms, including inoculum dilution, the creation of physical barriers constraining pathogen transmission, and amelioration of pathogen pathogenicity, fungicide resistance, and evolution [85,86]. Crop diversification also improves soil fertility [87] and microbial diversity, which in turn enhances nutrient availability for rigorous crop growth and microbial complexity to compete with pathogens [88]. In wheat, the take-all disease caused by *Gaeumannomyces graminis* (Sacc) Arx and Olivier var. *tritici* is observed to be more severe in monoculture than in fields with diversified crops [89]. Similar patterns were found in Huanglongbing caused by *Candidatus* Liberibacter asiaticus [90] and brown patch caused by *R. solani* [91] in citrus and turfgrass.

## 4. The Natural and Economic Considerations of Plant Disease Management with Biological Control Agents

**Figure 1:**
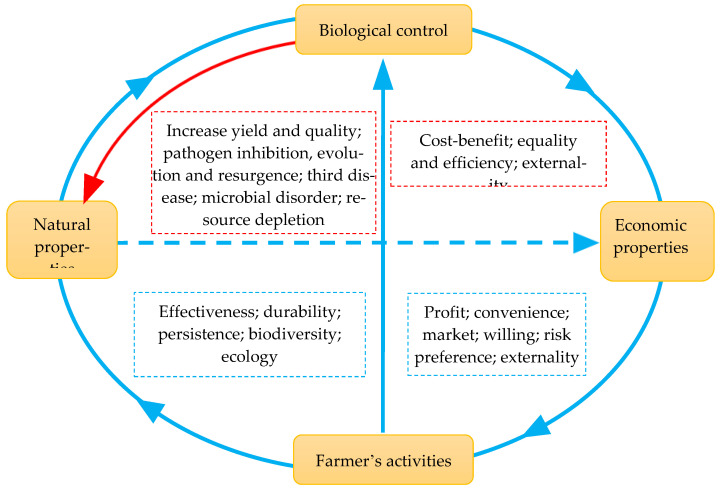
A diagram showing the interconnection of natural and economic properties of farmer adoption for biological control. Biological control of plant diseases can generate multifaced effects, including natural (e.g., pathogen inhibition, evolution, the third-party epidemics, nutrient supply, plant growth support, and resistance against biotic and abiotic stresses, saving yield and quality) and economic (e.g., cost, efficiency, benefit, externality) properties. In turn, these properties, particularly economic profit, convenience, and supply–demand market of technology and products, determine the choice of farmers in using biological control and other agricultural practices through the adaptation of their willingness, risk preferences, and expectations.

Biological control can generate multiple effects in food production, nutrient supply, and environmental health, thereby affecting economic development and ecological sustainability (Figure 1). The BCAs must be effective to give high crop yields and good crop quality and provide an economic incentive to the end-users compared to other disease management approaches. Ideally, BCAs should positively contribute to ecosystem services, such as improving soil fertility and biodiversity for succeeding agricultural production. In reality, the ability to identify the balance between the natural ecosystem and economic stability remains a hurdle because of a complex interaction among players, but this is essential for the commercialization of biological control approaches.

### 4.1. Effectiveness

Effectiveness is the primary consideration of biological control. BCAs must have a visual impact on disease epidemics either by suppressing pathogen growth or promoting host immunity to ensure crop yield and quality with a good economic return. The commercial BCAs of *Fusarium* wilt in lentils reduced disease incidence up to 50.0% and increased yield up to 58.7% [92]. *B.* *myloliquefaciens* (VFS2, VF11, VFS14, VFS15, and VFS21) significantly suppressed *Fusarium* *equiseti* (Corda) Sacc. disease in *Vicia faba* plants, reducing disease up to 100% and increasing plant growth up to 82% [93]. The effectiveness of BCAs is highly associated with their modes of action [94] and often has tradeoffs with other natural properties of the agents, such as their specificity as well as persistence in environments [43,95]. Therefore, it is of utmost importance to consider the genetics, biology, and evolutionary potential of pathogens in an ecological context. BCAs should exhibit self-regulation in their reproduction and can track the density and spatiotemporal distributions of pathogens such as auxin, which is powered by feedback-regulated mechanisms with self-organizing properties capable of generating highly context-specific responses to signal stimulation [96]. BCAs of *Botrytis* bunch rot reduced disease severity by 21–58% and maintained biological control activity for several seasons in vineyards [97]. Many BCAs are very vulnerable to fluctuations of the biotic and abiotic environment [95]. Their effectiveness in laboratory evaluation cannot always translate to in vivo effectiveness [79,98]. For example, many *Pseudomonas* BCAs show good performance in trials but cannot translate into consistent, effective disease management in diverse field situations [99]. For BCA *Candida oleophila* Montrocher strains against *Penicillium expansum* Link of apple disease, a significant difference in enzymatic activity existed between in vivo and in vitro application [100]. Therefore, it is important to select the agents that have stable effectiveness under various environmental conditions, such as soil texture, moisture, temperature extremes, or competition.

### 4.2. Durability

Pathogens empower the ability to evolve in response to environmental changes. Continual applications of the same BCAs on a commercial scale could pose a strong selection of pathogens, which may eventually lead to the emergence of new pathogen populations able to escape or mitigate the adverse effects of the BCAs [101]. This adaptability of pathogens is developed in an eco-evolutionary process involving many biotic and abiotic factors in the ecosystems, including the genetics and the biology of target pathogens, and BCAs, as well as community structure [81,102,103] and ongoing climate changes, may complicate the interaction further, which is favorable to the evolution of pathogens to evade the BCAs currently used. Although rarely reported in practice, erosion of BCAs against plant pathogens has been documented in laboratory and greenhouse conditions. The BCA effects against *B. cinerea* disease of *Astilbe hybrida* significantly reduced after eight successive treatments and were totally lost after ten treatments [104]. The sensitivity of *B. cinerea* to pyrrolnitrin, an antibiotic derived from many BCAs, was rapidly reduced after 10 generations of passage [105].

The durability of BCAs is negatively associated with the evolvability of target pathogens [106]. Shorter durability is expected in the BCAs targeting pathogens with higher genetic variation from sexual reproduction, large effective population size, and long dispersal ability compared to those with lower genetic variation, clonal reproduction, small effective population size, and limited dispersal ability [107]. The durability of BCAs also depends on their modes of action and how they are applied in agriculture [17,108]. BCAs with a higher specificity are expected to be overcome more easily and are therefore relatively less durable than those with a lower specificity due to strong natural selection [107]. Like fungicides, BCAs with multiple modes of action are expected to be most durable because of the requirement of multiple mutations accumulated in the pathogen genome for the development of resistance [17,101,109,110].

### 4.3. Ecological Sustainability

An ecosystem is linked by the interaction of species with their physical environment through nutrient cycles and energy flows. It undergoes consistent dynamics spatially and temporally in response to the change of any individual biotic and abiotic components. An introduction of BCAs to the interactions either through the applications of living organisms or compounds to farmlands inevitably modify the compositions and functions of the entire ecosystem temporarily or permanently. Good BCAs should not only be effective to suppress disease epidemics for high crop yield and quality in the current production but also do not have deleterious impacts on ecosystems supporting future agriculture and socioeconomics. This could be achieved by enhancing natural enemy/competitors, beneficial species, and ecological efficiency by nurturing functional biota [111]. This concern of ecological safety has led to a heated debate between conservationists and practitioners, particularly in western countries, and a clouded approach to control plant diseases. Ecological philosophers argue that biological control may lead to species extinction in extreme cases, threatening ecological function and resilience [112], but agricultural pragmatists claim there is no evidence of such impacts [113].

Because of the ecological and economic impacts of invasive species on natural sustainability and societal development [114], there is also a concern that BCAs may eventually become an invasive species, causing permanent damage to local ecosystems, as documented outside the field of plant pathology, such as using hares to control grass in Australia [115] and *Harmonia axyridis* Pallas to control aphid populations in the USA [102,116]. These phenomena mainly occur in systems involving the use of animals and insects as BCAs [117]. The lack of natural enemies in the new environments provides them with a unique opportunity for survival and reproduction. Although the same events have been rarely reported in plant pathology involving the use of beneficial microorganisms, the possibility cannot be excluded. These BCAs may become an emerging pathogen of local crops due to host-jumping or other eco-evolutionary events. Therefore, candidate BCAs should be thoroughly evaluated to understand the biological, ecological, and evolutionary outcomes before they are introduced for commercial application.

### 4.4. Economic and Practical Incentives

Farmers decide what technologies they use to control plant diseases, and their attitude to biological control is powered by economic, technological, and practical factors such as effectiveness, profit, availability, and convenience [114,118]. To attract farmers’ adaptation of biological control, technology should be easy to assess, ready to use, and lead to economic advantages relative to others in terms of the supply and demand relationship and cost efficiency (Figure 1). For example, formulations in granules that can be stored at room temperatures and applied by simple machines are generally favored by farmers in developing countries as opposed to those requiring more expensive equipment or refrigerator storage [119]. The lack of understanding of the technology features and fewer choices of successful technologies discourage farmers from adopting the biological control approach. In this case, information exchange between technology developers and end-users through training and field demonstration is important.

Apart from the direct impact on the expenses and income of farmers, biological control also indirectly influences their economic benefits, called externality, through its effect on ecological function and sustainability in the farmlands [80,83,84,114]. This externality, although not directly affecting the cash flow of farmers in the immediate term, should be reflected in the calculation of net profit in using BCAs. From a broader perspective, good BCAs also generate economic impacts on society because their positive externalities reduce the social costs to retain a functional ecosystem, while negative externalities resulting from pitfalls of biological control inevitably escalate the financial burden of society necessary to restore the damaged ecosystems [80,103]. To fully understand the economic benefits of biological control, a comprehensive evaluation of its natural and societal functions, social (public) and individual (farmer) benefits, and long- and short-term impacts are required [118]. Farmers’ attitudes to biological control can be promoted by the internalization of positive externalities through governmental and public incentives of financial supports.

## 5. Enhance Efficiency of Biological Control through Eco-Evolutionary Principles

The paradigm of biological control, like other approaches of plant disease management, emphasizes the importance of immediate impact on disease reduction but overlooks its long-term interactions with pathogens, the ecosystem, and society. As mentioned earlier, disease epidemics in modern agriculture are largely associated with a disrupted ecosystem driven by inappropriate practices, including disease management [80], which may unconsciously aggravate the disease or the third-party epidemic [103]. Thus, biological control should stress the importance of multidisciplinary efforts on the basis of the IDM principle and shift its philosophy from single disease management to better serve both nature and society. The ethical and social functions should also be considered in the choice and execution of biological control. Here, we formulate a framework of biological control guided by the eco-evolutionary principle to enhance its effectiveness, durability, efficiency, ecological safety, and economic incentive to sustainably serve both society and nature.

### 5.1. The Development of “Green” and Cost-Effective Biological Control Agents

Despite the promising perspective of using the biological control approach, many farmers, particularly in developing countries, are reluctant to seek the practice either due to its technical infirmity, ecological consideration, or economic attractiveness [83,101,113,120]. There is also an urgent need to develop a new generation of BCAs to meet increasing societal demand for more production of high-quality and diverse foods with “green” effects on ecosystems [81,103]. Many BCAs used currently act either as an antagonism against pathogens or as a promotor to increase host immunity [13,26,36,50,55]. The first category may be very effective at the beginning, but its effectiveness can be lost quickly due to the evolution of pathogens. The second category may be more durable but less effective due to the time lag to induce host immunity unless it is applied sufficiently early before pathogen attacks [79]. Future attention should be directed to the invention of green BCAs with regulatory effects on ecosystems, such as the use of microbiome- or conservation-based approaches centering on the synthetic function of efficacy, durability, persistence, complementation (with other approaches), and sustainability. Technological advantages in molecular screening and knowledge accumulation in genetic and evolutionary mechanisms of ecological functions make the development possible.

When developing new agents, the biology and the evolutionary adaptation of pathogens should be considered [121] together with the mode of action of the BCAs, and the evaluation of their synthetic functions should be conducted at a pathogen-metapopulation level composed of strains of various geographic and genetic origins and under various ecosystems to optimize outcomes. Cost-effectiveness for mass-production and application is also an overriding factor to be considered during screening [122]. Many BCAs, particularly those involving beneficial microorganisms, are sensitive to environments. Temperature and relative humidity are two main environmental factors regulating the efficacy of BCAs, as documented in the observed response of *Botrytis* bunch rot to several commercial BCAs [123]. Therefore, it is important to evaluate and BCAs according to specific climate conditions, community composition, host and pathogen populations as well as farming practices [95]. Therefore, it may be necessary for a single BCA to be formulated in different forms or together with BCAs to meet the requirements of a particular ecosystem [124].

### 5.2. Use Together with Other Disease-Control Approaches

A successful expression of BCAs depends on environmental conditions [125]. A BCA can be used together with other disease management approaches to enhance efficacy [126]. For example, alternations or mixtures of BCAs with fluopyram increased efficacy against grey mold on bunches by ~10% and 60% compared to chemical and BCA strategies alone, respectively, and reduced the spread of SDHI-resistant phenotypes [127]. This benefit likely results from complementary or enhancement effects of different disease-control agents [128]. Attempts should be made to combine BCAs with other agents possessing different modes of action. Among them, crop genetics, pathogen population survey, and disease forecast are the most promising partners [76,95,129]. Many BCAs need time to establish on the host surface or root rhizosphere; disease forecast according to the conduciveness of local climate conditions and plant genetics can synchronize application time and frequency of BCAs with the developmental dynamics of plant diseases, which allows the former to colonize and take effects. This integration has been documented in the control of *Botrytis cinerea* Pers diseases of tomato and cucumber [129]. On the other hand, population surveys provide knowledge on pathogen compositions in farmlands [76], which can be used to determine appropriate BCAs for maximum control [43,76].

Plant health status, microbiome, and ecological diversity also affect the efficacy of BCAs [130,131,132,133]. In this case, practical models to improve soil fertility, such as the availability and balance of nutrient and microbial diversity through agricultural diversification, should be a primary concern. It has been shown that the efficacy of using parasitoids to control *Cucumber mosaic virus* and *Cucurbit aphid-borne yellow virus* vectors was substantially reduced in fields located at simplified landscapes compared to complex landscapes [132,133]. Optimizing resource allocation to coincide with plant phenology through the regulation of resource availability by rational water and fertilizer applications, cropping system adjustment, and/or cultivation intensification can improve the efficacy of biological control [131]. Thus, we should pay attention to community structure and composition, such as interspecific relationships, spatial heterogenicity, and genetics and physiology of plants, in the application of BCAs.

### 5.3. Formulate a Dynamic Disease Management Program

Plant pathogens undergo rapid and continuous evolution in response to environmental stresses, such as those posed by climate change and disease-control approaches [27,134,135,136,137]. A major challenge associated with the commercialization of BCAs is the development of resistance in pathogen populations, and this phenomenon has been recorded in several pathogen–BCA interactions [104,105,108]. To achieve a durable efficacy, it is important to formulate biological control strategies in an adaptive way by the dynamic use of BCAs spatially and temporarily according to the population genetics and evolutionary ecology of host–pathogen–BCA interaction [17,110]. This dynamic approach creates a diversifying selection against pathogen populations to prevent the emergence of mutants with the ability to overcome the BCAs. Cocktails have been used to conserve the efficacy of bacteriophages because diversified selection generated by different phages can limit the target bacteria to developing resistance against all of them [108]. BCAs statically used in space and time are expected to be overcome by pathogens more easily, and therefore are less durable relative to those dynamically used—the phenomenon has been widely documented in host resistance genes and fungicides [17,107,110]. In addition, dynamic and adaptive deployment may also create an impact on an ecosystem supporting higher biodiversity, which generates positive feedback on the durability and efficiency of the BCAs [29,132,133]. This dynamic approach should be formulated in the context of ecology and evolutionary principles, and evolutionary biologists should play an important role in the formulation. The dynamic program can also be used in combination with other plant disease management programs to further improve efficacy and durability (see Section 5.2).

## 6. Social Involvement

Regardless of its effectiveness, biological control is not considered to be successful if it is not adopted by local communities. Thus, efforts are needed to understand the attitudes and perceptions of communities on biological control [138]. Such efforts must include an educational component that involves the awareness of government, the public, and farmers in the eco-evolutionary concept and perspective of sustainable disease management and inform community members about the natural and societal benefits of using BCAs for sustainable disease management as an alternative to other approaches, such as synthetic chemicals, which may be more effective and easier to apply but more harmful to humans, livestock, and environments in the long run [139]. Outreach efforts can also include the training of community members on how to safely use BCAs and the roles of government in the regulation and research development of BCAs. It is a rule of thumb that before commercial approval, BCAs are strictly evaluated for their safety for human, animal, and environmental exposure [139]. While large companies in developed countries can profit from the final products, this is not always the case for companies in developing countries, discouraging research and development on these potential promising disease-management strategies. In such cases, financial supports for research development and initial application are required.

## 7. Concluding Remarks

Historically, the demand for high crop yield has been met by pesticides application, resistance gene deployment, and other approaches. However, the comprehensive benefit of these approaches has been challenged either due to their ecological impacts or long-term effectiveness [83]. Among the alternatives, biological control appears to be one of the most promising approaches for eco-friendly and sustainable agriculture to protect crop plants and safeguard food [120]. Nevertheless, currently, it is insufficient to draw a conclusion that the application of biological control could actually improve the efficiency, profit, and convenience of disease management and agricultural productivity [101]. Unexplored aspects for further research remain, including information of the mechanisms, technological traits, and farmers’ applications related to natural and economic perspectives, reactions with other agricultural practices, durability, and the ecological evolution principle.

Economic attractiveness and technical availability are essential to incite farmers to apply biological control approaches. There is much need for the provision of feasible and affordable BCAs, education, and policy support. Complex interactions among crop plants, pathogens, BCAs, and physical environments may lead to biological invasion, threatening the local ecosystem and increasing the risk of erosion or complete loss of the efficacy of biological control approaches if they are used in a single and static manner for a long term. This highlights the importance of applying the ecological evolutionary principle to synergically evaluate the efficacy, efficiency, durability, and environmental safety of biological control. Therefore, efforts should be made to build concepts to understand the ecological tolerance, social acceptability, and economic accessibility of biological control strategies.

## Data Availability

Not applicable.
